# Optimisation of immunofluorescence methods to determine MCT1 and MCT4 expression in circulating tumour cells

**DOI:** 10.1186/s12885-015-1382-y

**Published:** 2015-05-10

**Authors:** Stephen Kershaw, Jeffrey Cummings, Karen Morris, Jonathan Tugwood, Caroline Dive

**Affiliations:** Clinical and Experimental Pharmacology Group, Manchester Cancer Research Centre, Cancer Research UK Manchester Institute, University of Manchester, Manchester, M20 4BX UK

**Keywords:** Monocarboxylate transporters, MCT1, MCT4, Biomarkers, Immunofluorescence, Circulating tumour cells, Optimisation, AZD3965

## Abstract

**Background:**

The monocarboxylate transporter-1 (MCT1) represents a novel target in rational anticancer drug design while AZD3965 was developed as an inhibitor of this transporter and is undergoing Phase I clinical trials (http://www.clinicaltrials.gov/show/NCT01791595). We describe the optimisation of an immunofluorescence (IF) method for determination of MCT1 and MCT4 in circulating tumour cells (CTC) as potential prognostic and predictive biomarkers of AZD3965 in cancer patients.

**Methods:**

Antibody selectivity was investigated by western blotting (WB) in K562 and MDAMB231 cell lines acting as positive controls for MCT1 and MCT4 respectively and by flow cytometry also employing the control cell lines. Ability to detect MCT1 and MCT4 in CTC as a 4^th^ channel marker utilising the Veridex™ CellSearch system was conducted in both human volunteer blood spiked with control cells and in samples collected from small cell lung cancer (SCLC) patients.

**Results:**

Experimental conditions were established which yielded a 10-fold dynamic range (DR) for detection of MCT1 over MCT4 (antibody concentration 6.25 μg/mL; integration time 0.4 seconds) and a 5-fold DR of MCT4 over MCT 1 (8 μg/100 μL and 0.8 seconds). The IF method was sufficiently sensitive to detect both MCT1 and MCT4 in CTCs harvested from cancer patients.

**Conclusions:**

The first IF method has been developed and optimised for detection of MCT 1 and MCT4 in cancer patient CTC.

## Background

A common trait exhibited amongst cancer cells is increased glucose consumption and a switch from regular oxidative phosphorylation to glycolysis, which results in rapid proliferation and an increased malignant phenotype [1-3]. The monocarboxylate transporters (MCTs) are a group of membrane-bound proteins that facilitate the proton-coupled co-transport of lactate across the plasma membrane [4,5]. In glycolytic tumours, the MCTs are responsible for the efflux of lactate and promotion of an acidic extra-cellular environment, both of which contribute to tumour metabolism and a malignant phenotype [4,6,7]. Hence, inhibition of MCT may provide a selective method of eliminating glycolytic cancer cells [8-10].

AZD3965 is a novel inhibitor of the predominant MCT isoform (MCT1) and prevents the release of lactic acid by hypoxic/glycolytic tumour cells, causing accumulation of acidic lactate within the tumour cell, and inhibition of cell growth and survival [8,11]. MCT1 is also responsible for the uptake of lactate in normoxic tumour cells, which converts the metabolite into pyruvate as an energy source [7]. Hence, inhibiting lactate uptake increases the dependency of aerobic tumour cells on glucose, and as a consequence deprives hypoxic cells of glucose precipitating their cell death [5,7]. However, it has also been shown that the presence of other MCT isoforms (notably MCT4) continues the transport of lactate despite MCT1 inhibition and thus could act as a potential mechanism of drug resistance to AZD3965 [8,11]. Therefore, for a more comprehensive mechanistic-based evaluation of the drug, it is advantageous to profile circulating tumour cells for MCT1 and MCT4 expression to elucidate the association between these transporters and AZD3965 sensitivity or resistance.

Recruitment has now commenced in the UK at the Royal Marsden and Freeman Hospitals in a Phase I Trial of AZD3965 in patients with advanced cancer (NCT01791595/CRUKD/12/004, http://www.cancerresearchuk.org/cancer-help/trials/a-trial-azd3965-for-advanced-cancer). In this Cancer Research UK-funded trial, an emphasis is being placed on providing both proof of mechanism and proof of principle by the incorporation of pharmacodynamic and predictive biomarkers (http://clinicaltrials.gov/show/NCT01791595). We describe the development and optimisation of an immunofluorescence (IF) assay for the detection of MCT1 and MCT4 in cancer patient CTCs employing the Veridex™ CellSearch system. Our studies utilised control cancer cell lines known to only express MCT1 (K562) or MCT4 (MDAMB231) [12] and focused on assay optimisation. The ability to detect MCT1 and MCT4 in CTCs harvested from cancer patient blood specimens was confirmed. The goal is to apply the new IF methodology as potential prognostic and predictive biomarker assays in the on-going clinical trials of AZD3965 (and other compounds in this class).

## Methods

### Western blotting of MCT1, MCT4, cytokeratin, and EpCAM antibodies in control cell lines

Pan-cytokeratin (Thermo Scientific, Clone C-11, #4545), EpCAM (Thermo Scientific, Clone VU1D9, #MS-144-P) antibodies, as well as MCT1 (H-1) (Santa Cruz, sc-365501) and MCT4 (H-90) (Santa Cruz, sc-50329) antibodies both of which were custom conjugated with the AF488 fluorophore by Santa Cruz, were used to characterize the K562 and MDAMB231 cell lines as appropriate controls. K562 cells (ATTC CCL-243) were grown in RPMI (Gibco) supplemented with 10% foetal bovine serum (FBS, Gibco) and MDAMB231 cells (ATTC HTB-26) were grown in DMEM (Gibco) supplemented with 10% FBS (Gibco) and 2 mM L-Glutamine (Lonza). All repeat *in vitro* experiments were conducted on cells within a maximum of 5 passages. Adherent cell cultures were harvested by treatment with Accutase dissociation solution (Sigma Aldrich, #A6964) for 1–3 minutes at 37°C and then re-suspended in growth medium. After centrifugation, cells were washed in 10 mL ice-cold PBS, and re-suspended in 100 μL of PBS supplemented with 1% protease inhibitor cocktail (Cell Signaling, #5871S), 1% phosphatase inhibitor 2 (Sigma Aldrich, #P5726), 1% phosphatase inhibitor 3 (Sigma Aldrich, #P0044), and 10% Cell Lysis Buffer (Cell Signaling, #9803). The cells were left to lyse on ice for 30 minutes with intermittent agitation to ensure cell debris did not clump. The lysates were centrifuged at 13000RPM for 10 minutes (using the Heraeus Sepatech Biofuge 13 microfuge) at 4°C and the supernatant collected for protein concentration determination by BCA protein assay according to the kit instructions (Pierce). The fluorescent output of cell lysates were measured alongside known BCA standards (using the Labsystems Original Multiskan EX plate scanner) and the protein concentrations were determined through interpolation against the BCA standard curve. The lysate protein concentrations were subsequently diluted to 1 mg/mL in 1x Laemmli buffer (6 mL double-distilled water + 4% SDS + 20% glycerol + 10% 2-mercaptoethanol + 0.004% bromophenol blue + 0.125 M Tris HCl; adjusted to pH6.8).

1 mg/mL of K562 and MDAMB231 lysates were boiled at 100°C for 10 minutes and left to electrophorese on a 12% acrylamide gel at 150 V for 90 minutes using 1x Running Buffer (30 g Tris + 144 g glycine + 10 g SDS + 1 L double-distilled water) and then transferred onto PVDF membranes at 100 V for 60 minutes using 1x Transfer Buffer (200 mL 10x Transfer Buffer + 400 mL Methanol + 1.4 L double-distilled water). All western blots were performed on Invitrogen Mini Cell and Bio-Rad Mini Protein Apparatus using the Bio-Rad 034BR power pack. The PVDF membranes were blocked in PBS containing 0.1% Tween-20 (PBST) + 5% milk (Marvel Original Dried Milk Powder) for 60 minutes at room temperature prior to overnight incubation with the primary antibodies all diluted to 2 μg/mL (1:1000 dilution in PBST + 5% milk at 4°C. Membranes were washed three times in PBST and left to incubate with the appropriate secondary antibodies (goat anti-rabbit or goat anti-mouse IgG/HRP-conjugated, DAKO) all diluted to 0.05 μg/mL (1:5000 dilution in PBST + 1% milk) for 60 minutes at room temperature. Membranes were washed in PBST, placed in a 1:1 solution of oxidising ECL reagent and luminescent ECL reagent (Western Lighting Plus-ECL) and visualised under chemiluminescence for the detection of protein bands at the expected molecular weights (using the Fujifilm Intelligent Dark Box II). Membranes were then left to incubate with GAPDH (Abcam, #ab9485) and a goat-anti-rabbit IgG/HRP secondary antibody (DAKO) as a protein loading control, both for 60 minutes at room temperature, and visualised using ECL reagent as stated above.

### Optimisation of MCT1/MCT4 antibody concentration as 4^th^ channel markers using flow cytometry in control cell lines

K562 and MDAMB231 cells were detached with Accutase solution as above, centrifuged at 1200RPM for 5 minutes at room temperature (using the CWS Anita II PK121 centrifuge), and re-suspended to 1-5×10^6^ cells/mL with FACS staining buffer (100 mL PBS containing 1% bovine serum albumin [BSA] and 0.1% sodium azide). The cells were left to fix and permeabilised in 250 μL FACS fixation/permeabilization buffer (BD Biosciences; #554714) at 4°C for 20 minutes with intermittent agitation to ensure proper fixation of individual cells. The cells were washed three times in 1x permeabilisation/wash buffer, re-suspended in 100 μL of serial dilutions of the primary AF488-conjugated MCT1 (Santa Cruz, #sc-365501), MCT4 (Santa Cruz, #sc-50329), Normal Rabbit isotype control (Santa Cruz, #sc-45068), and Normal Mouse isotype control (Santa Cruz, #sc-3890) antibodies and left to incubate at 4°C for 60 minutes with intermittent agitation to ensure a homogenous cell suspension. The cells were then washed in 1x permeabilisation/wash buffer and re-suspended in FACS staining buffer and analysed using the BD 3-Colour FACSCalibur II system.

### Detection of MCT1/MCT4 by Veridex™ CellSearch system in control cell lines spiked into healthy normal volunteer (HNV) whole blood

Blood was also collected from healthy normal volunteer (HNV) donors for cell spiking experiments according to a local ethics committee-approved protocol (NHS North West 9 Research Ethical Committee, Manchester, UK) and in compliance with the Helsinki Declaration (http://www.wma.net/en/30publications/10policies/b3/index.html). K562 and MDAMB231 cells were detached using Accutase solution and spiked into 7.5 mL of HNV blood (collected as previously stated) at a final cell count of 200–300 per mL. 6.5 mL of Veridex™ Dilution Buffer was added to each tube, inverted, and centrifuged at 800 x g for 10 minutes at room temperature prior to automated processing on the Veridex™ CellSearch system. The spiked K562 and MDAMB231 cells were enriched by EpCAM-targeted magnetic nanoparticles and left to incubate with CK-PE (Veridex™), DAPI (Veridex™), CD45-APC (Veridex™), 6.25 μg/mL of MCT1-AF488 (Santa Cruz, #sc-365501), and 8 μg/100 μL of MCT4 AF-488 (Santa Cruz, #sc-50329) antibodies before being transferred into MagNest cartridges for subsequent immunofluorescent imaging. The MagNest cartridges were scanned using the Veridex™ CellSearch Analyzer II at a range of different exposure times to optimise the appropriate time for sufficient MCT1/MCT4 visualization in the 4^th^ channel.

### Detection of MCT1/MCT4 by Veridex™ CellSearch system in CTC harvested from blood specimens collected from in SCLC patients

Blood samples for CTC enrichment and enumeration were collected from a total of 2 SCLC patients with written informed consent being obtained from both patients entered into an experimental medicine study being conducted at the Cancer Research UK Manchester Institute and in compliance with the Helsinki Declaration (http://www.wma.net/en/30publications/10policies/b3/index.html). The study was prospectively approved by the NHS North West 9 Research Ethical Committee, Manchester, UK. Samples were taken into CellSave tubes (Veridex™), containing EDTA and a cellular preservative and maintained at room temperature for no longer than 96 hours prior to analysis. 7.5 mL of patient blood was added to 6.5 mL of Veridex™ Dilution Buffer, centrifuged, and processed using the same antibodies and procedures as previously stated above. The resulting MagNest cartridges were scanned at 0.4 seconds (for MCT1) and 0.8 seconds (for MCT4) as optimised during cell spiking experiments.

## Results

### Characterisation of control cell lines and evaluation of antibody selectivity by western blotting

Expression of both CK and EpCAM in the two control cell lines - K562 and MDAMB231- is essential to ascertain their suitability for utilisation in future experimentation. EpCAM is required for sequestration of CTC from blood by the Veridex™ CellSearch system by immunomagnetic isolation, and CK for staining and visualisation of the isolated cancer cells. Conversely, due to the reported specificity of expression of MCT1 and MCT4 in K562 and MDAMB231 respectively, these cell lines were also employed to evaluate antibody selectively [12].

**Figure 1:**
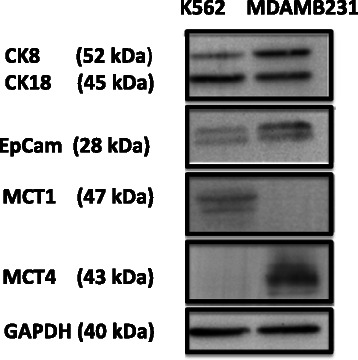
Western blots of cytokeratin (CK)-8 and −18, epithelial cell adhesion molecule (EpCAM), monocarboxylate transporter-1 (MCT1), monocarboxylate transporter-4 (MCT4), and glyceraldehyde 3-phosphate dehydrogenase (GADPH) in K562 and MDAMB231 lysates. Protein lysates were loaded at a concentration of 1 mg/mL into each lane of the western blot. Molecular weights of each protein were determined using ImageJ software, with GAPDH staining serving as a control for the loading of protein within each well of the western blot.

Figure 1 shows that both cell lines chosen expressed protein bands corresponding to the molecular weight of CK-8 and CK-18, known to be detected by the pan-cytokeratin antibody used in the present study [13]. Bands were also evident in both cell lines corresponding to the dimerization products of EpCAM, indicative of protein degradation after detachment with Accutase enzymatic solution [14]. Thus, the two control cell lines expressed the two proteins required to be successfully enriched and detected by the Veridex™ CellSearch system. Protein bands were apparent at the expected molecular weight of MCT1 (47 kDa) in K562 but absent in the MDAMB231 (see Figure 1), as previously published [12]. While, bands were detected at the expected molecular weight of MCT4 (43 kDa) in MDAMB231 but absent in K562 (Figure 1), again confirming previously published studies [12]. Thus, the two cells K562 and MDAMB231 appear to be good candidates as positive and negative controls in cell spiking experiments to optimise the selective detection of MCT 1 and MCT4 in CTC harvested from cancer patients by the CellSearch system.

### Optimisation of staining intensity

Flow cytometry was employed together with the two control cell lines in order to maximise the dynamic range of the IF assays to detect MCT1 and MCT4 in cancer cells. A range of antibody concentrations were investigated alongside negative controls (unstained cells) and species-specific isotype controls. The optimum antibody concentration was determined as that producing the greatest fold-increase in MCT1/MCT4 mean fluorescence intensity (MFI) whilst retaining the lowest percentage of non-specific background fluorescence. Unstained cells were used as a negative control to account for the presence of innate auto-fluorescence within each control cell line. The isotype control was an additional antibody that was raised in the same species as the primary antibody (e.g. mouse or rabbit) and used at the same concentrations to confirm that the primary antibody binding is specific to its target and not due to the interaction with Fc receptors or other proteins.

**Figure 2:**
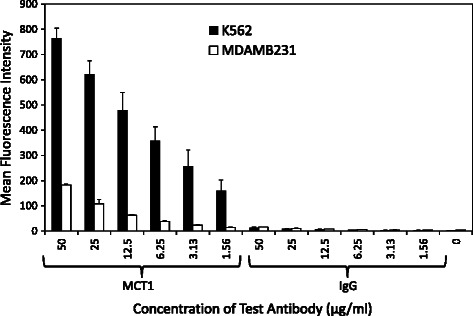
Average Mean Fluorescence Intensity (MFI) of the MCT1 and species-specific isotype (mouse IgG) control antibodies in K562 and MDAMB231 cells. The results are represented as the averaged mean fluorescence intensity (geometric mean) plotted against the antibody of interest (n = 3), with error bars representing the standard deviation across triplicate data sets for each antibody concentration.

Flow cytometric analysis of cells stained with MCT1 antibody demonstrated a significant increase in MFI across the range of antibody concentrations utilised both in K562 and to a much lesser extent in MDAMB231 (Figure 2). Here, the fluorescence intensity emitted by K562 cells was on average 5–10 fold greater than that emitted by MDAMB231 cells and well in excess of that detected in unstained controls and species-specific isotype controls (data not shown). These data are consistent with the fluorescence signal being attributable to an interaction of the primary antibody with its target protein MCT1 and not due to auto-fluorescent cells or off-target Fc receptor binding. Analysis of the averaged MFI values revealed that an antibody concentration of 6.25 μg/mL produced the greatest fold-increase in MCT1 fluorescence (9-fold) whilst retaining the lowest percentage of non-specific background fluorescence (10%). Consequently, 6.25 μg/mL was selected as the optimum concentration of MCT1 antibody for cell spiking experiments using K562 and MDAMB231 cells.

**Figure 3:**
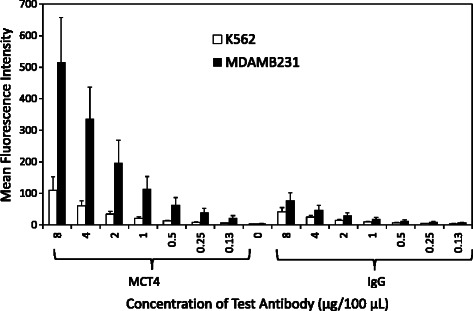
Average MFI of MCT4 and species-specific (rabbit IgG) antibodies in K562 and MDAMB231 cells. The results are represented as the averaged mean fluorescence intensity (geometric mean) plotted against the antibody of interest (n = 3), with error bars representing the standard deviation across triplicate data sets for each antibody concentration.

Flow cytometry of K562 and MDAMB231 cells stained with MCT4 antibody demonstrated a similar profile of MFI across all antibody concentrations investigated analogous to the studies with the MCT1 antibody. The level of this fluorescent signal also significantly surpassed that measured in both the unstained controls and species-specific isotype controls and is thus considered due to a specific interaction of the antibody with is target protein MCT4 (Figure 3). Here, an antibody concentration of 8 μg/100 μL produced the greatest fold-increase in MCT4 fluorescence whilst maintaining a low degree of background fluorescence. This concentration was therefore selected as the optimum for cell spiking experiments using K562 and MDAMB231 cells.

Nonetheless, in both Figures 2 and 3 there was a modest increase in signal in the negative control dependent on antibody concentration, that was effectively absent in the IgG control. This raises the possibility that MCT1 and MCT4 are not totally absent in MDAMB231 and K562 cells, respectively. However, in previous studies the selective expression of MCT1 and MCT 4 in was K562 and MDAMB231 was confirmed by siRNA [12].

### Optimisation of CellSearch system operating parameters for determination of MCT1 and MCT4 in control cell lines spiked into volunteer blood

Defined numbers of cells from the 2 control lines (K562 and MDAMB231) were added to HNV blood in triplicate for analysis by the CellSearch system employing instrumental methods similar to those used routinely in the isolation and enumeration of CTCs from cancer patient blood samples. The CellSearch system uses anti-cytokeratin-phycoerythrin (PE) to label the intracellular protein cytokeratin (specific for epithelial cells), 4′-6-diamino-2-phenylindole (DAPI) to label the cell nucleus, and anti-CD45-allophycocyanin (APC) to label leukocytes separate from the CTC population [15]. An additional channel on the CellSearch Analyzer II instrument is used for a user-defined marker; in this case; MCT1 and MCT4 AF488-conjugated antibodies. Thus, a CTC is defined as a cell that exceeds 5 μm in diameter and possesses a positive CK and DAPI signal, and a negative CD45 signal. These criteria were also used in the cell spiking experiments, to distinguish between cancer cells and circulating leukocytes in normal healthy volunteer blood.

Exposure/Integration time is a critical parameter associated with the CellSearch Analyzer II camera settings. The setting represents the amount of time that the shutter of the camera is open to allow light to pass through at a given wavelength.

**Figure 4:**
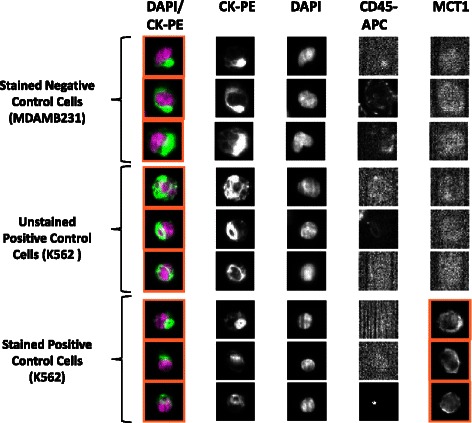
Optimisation of operating parameters for determination of MCT1 by immunofluorescence (IF) in control cell lines spiked into volunteer blood. The figure contains a gallery of images obtained by the CellSearch system in 4 different channels from a total of 9 different cells utilising discrete markers to characterise the cells. Images were generated at the optimum integration time of 0.4 seconds for detection of MCT1. Anti-cytokeratin-phycoerythrin (CK-PE) labels the intracellular protein cytokeratin (specific for epithelial cells), 4′-6-diamino-2-phenylindole (DAPI) the cell nucleus and anti-CD45-allophycocyanin (APC) labels leukocytes to allow discrimination between tumour cells. An additional channel contains a pseudo-coloured composite image of the DAPI and CK-PE stains. MCT1 was only detected in the 4^th^ channel in the positive control cell line K562 (see Figure 1) that had been stained with MCT antibody, but was absent in unstained K562 cells and the negative control cell (see Figure 1) MDAMB231 stained for MCT1.

**Figure 5:**
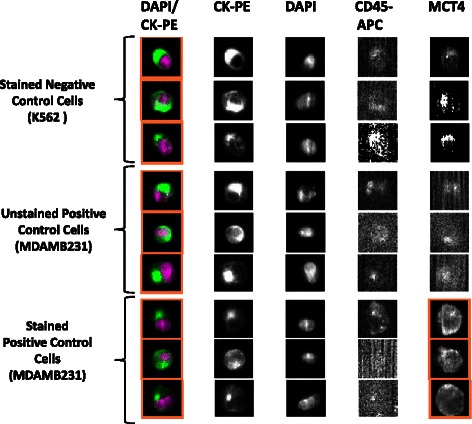
Optimisation of operating parameters for determination of MCT4 by immunofluorescence (IF) in control cell lines spiked into volunteer blood. The figure again contains a gallery of images obtained by the CellSearch system in 4 different channels from a total of 9 different cells. The images were generated at the optimum integration time of 0.8 seconds for detection of MCT4. Identification of the 4 single channels and the composite are as defined in Figure 4. MCT4 was only detected in the 4^th^ channel in the positive control cell line MDAMB231 (see Figure 1) that had been stained with MCT4 antibody, but was absent in unstained MDAMB231 cells and the negative control cell (see Figure 1) K562 stained for MCT4.

The integration time was optimized to detect the signal from the AF488-conjugated MCT1 and MCT4 antibodies, while still maintaining sufficient positive signal over background. All Veridex™ cartridges underwent a range of exposure times under fluorescent light (0.2, 0.4, 0.8, and 1.0 seconds, depending on the antibody) in order to determine the optimum value. The resulting images were analysed to determine the highest resolution with the least background staining. Unspiked HNV blood did not fluoresce in the 4^th^ channel when stained with the antibodies, therefore negating the possibility of background fluorescence in non-cancer cell populations within blood. The images presented in Figures 4 and 5 are selected representations of spiked cells stained with the MCT1 (Figure 4) and MCT4 (Figure 5) antibodies at the optimised integration time.

Selective detection of MCT1 was evident in the 4^th^ channel of the CellTracks Analyzer II system when using the optimal antibody concentration of 6.25 μg/mL. Furthermore, the fluorescence signal was attributable to both the presence of the target protein and MCT1 antibody (see Figure 4). From the range of integration times tested 0.4 seconds was sufficient for the visualisation of MCT1 while eliminating the trace background fluorescence seen in MDAMB231 negative control cells stained with MCT1 antibody (see Figure 1). Moreover, integration times does not appear to significantly affect the visualisation of MCT1 in high-expressing cells, yet produces significant changes in MCT1 visualisation in low-expressing cells. Figure 5 demonstrates that MCT4 visualisation was apparent in the 4^th^ channel of the positive control cells, whilst absent in the unstained and negative control cells at the optimal integration time of 0.8 seconds exposure. MCT4 images at 0.4 seconds exposure time have low resolution and no significant difference in image quality is evident between 0.8 and 1.0 seconds.

### Detection of MCT1 and MCT4 in CTCs isolated by the CellSearch from blood samples collected from SCLC patients

**Figure 6:**
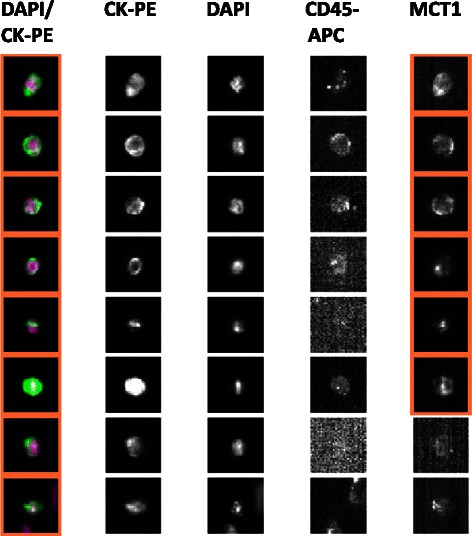
Confirmation that MCT1 can be detected in Circulating Tumour Cells (CTC) isolated from Small Cell Lung Cancer (SCLC) patients utilising the new immunofluorescence method developed in the present study. The gallery contains representative CTC harvested from 2 different patients. Operating parameters and identification of the 4 single channels and the composite are as defined in Figure 4. MCT1 staining in the 4^th^ channel is represented by heightened areas of fluorescence within the CTC cell membrane; diffuse staining seen in the cytoplasm can be described as a non-specific to MCT1.

**Figure 7:**
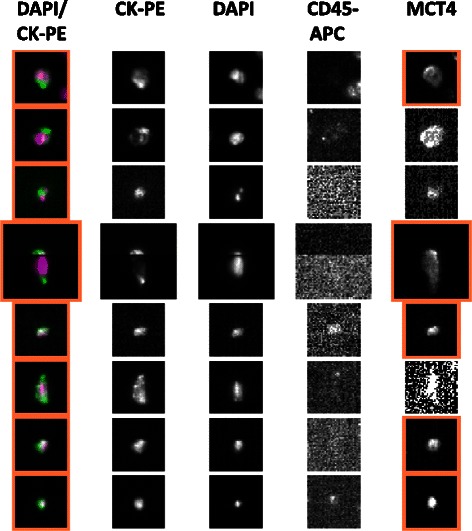
Confirmation that MCT4 can be detected in Circulating Tumour Cells (CTC) isolated from Small Cell Lung Cancer (SCLC) patients utilising the new immunofluorescence method developed in the present study. The gallery contains representative CTC harvested from 2 different patients. Operating parameters and channel identification are as defined in Figure 5. MCT4 staining in SCLC CTCs demonstrated a clear divide in expression levels between different cells of the same blood sample. Although the majority of SCLC CTCs expressed MCT4, a clear sub-population expressed MCT4 to minimal levels.

In the final experiment, blood specimens collected from cancer patients were analysed by the CellSearch system utilising the new IF labelling method and optimised instrumental parameters. The images presented in Figures 6 and 7 are examples of the CTCs isolated by the CellSearch system and stained for the MCT1 (Figure 6) and MCT4 (Figure 7). MCT1 staining was successful in enriched SCLC CTCs, of which a major proportion can be identified as expressing MCT1 within the cancer patient circulation. MCT1 staining in the 4^th^ channel is represented by heightened areas of fluorescence within the CTC cell membrane. Furthermore, as the MCT1 staining profile in the illustrated CTCs does not match the corresponding staining intensity observed in the DAPI channel, this signal is not considered nuclear. MCT4 staining in SCLC CTCs demonstrated a clear divide in expression levels between different cells of the same blood sample. Although the majority of SCLC CTCs expressed MCT4, a clear sub-population expressed MCT4 to minimal levels. Compared to the number of CTCs expressing MCT1, there appears to be a greater proportion that expresses MCT4, which is expected considering the hypoxic nature of SCLC tumours and the CTCs that disseminate from them.

In terms of the sensitivity of the CellSearch system to detect CTC in cancer patient blood, we have previously reported extensive validation studies on the precision and accuracy of CTC enumeration at low cell counts [16-18]. These studies employed advanced statistical techniques for data interpretation including β-expectation tolerance intervals (BETI) and β-content γ-confidence tolerance intervals. They were based on an analysis of blood samples collected from 38 different lung, prostate, melanoma and colorectal patients treated at the Christie Hospital, Manchester. With the aid of quality control procedures, a limit of detection of 1 to 5 CTC was achieved with only a total error of 30% determined by BETI analysis at β = 95%. Such values are within the expected and acceptable range for a typical biomarker assay, especially within the context of the fit for purpose approach [19-22]. Average recovery of 3 CTC from whole blood was 90% ± 9.6% coefficient of variation (CV) with total error of 28.3% determined by BETI analysis at β = 95%, while recovery of 25 CTC increased to 101% with a CV of 24% at a total error of 24.5% determined by BETI analysis at β = 95% [16].

## Discussion

The ISO 17025 international quality standard defines validation generically as the confirmation by examination and the provision of objective evidence that the particular requirements for a specific intended use are fulfilled. Such an approach -termed by many as fit-for-purpose (F-for-P) - has been embraced by translational scientists involved in clinical trials research, both in the pharmaceutical industry and academia, where compliance to GCP is often a statutory requirement [19,23]. F-for-P is particularly well suited to biomarkers assays since for a variety of reasons, the rigorous performance standards normally applied to bioanalytical methods are not appropriate [24,25]. During the present study where the aim was to optimise immunofluorescence methods for the selective detection of MCT1 and 4 in CTC isolated from cancer patient blood samples, F-for-P was adopted. A consistent body of data was obtained including western blot to characterise quality control cell lines, flow cytometry to optimise methodological parameters, volunteer studies and patient studies that supports the contention that the new methods are indeed fit for their intended purpose.

However, a difference in staining quality was observed between the two IF methods. Whereas, strong staining in positive K562 cells and an absence of staining in the negative MDAMB231 cells was evident in the MCT1 assay, a degree of background staining particularly in the negative K562 cells was observed with the MCT4 assay. This difference may be attributable to the polyclonal nature of the antibody used in the MCT4 assay, which is known to recognise a host of antigenic epitopes [26]. Notwithstanding, polyclonal antibodies can be inherently more sensitive than their monoclonal counterparts and amplify a low expression signal by binding to multiple epitopes on the target protein [26], which was observed in our western blot studies with cells lines (see Figure 1).

CellSearch remains the only CTC platform cleared by the FDA to monitor cancer patients [15] and the benchmark against which new technologies should be assessed [27]. As a biomarker, CTCs have been shown to correlate to survival in numerous cancer types [28-31] and to be predictive of response to targeted agents [32-35]. In relation to the AZD3965 trial, the opportunity to monitor MCT1 and MCT4 expression after drug treatment should add an extra dimension to CTC as a biomarker. For example resistance mechanisms to AZD3965 in patients may be explored by studying MCT4 expression.

However, despite the benefits of the CellSearch system, limitations have been identified notably the dependence on the expression of the epithelial maker EpCAM [18,29,36]. Thus, CTCs that do not express such a marker and/or have undergone the epithelial to mesenchyme transition (EMT) may escape detection. The net result is that MCT1 and MCT4 expression level may only be determined in a sub-set of cells that may not be representative of the total population of CTCs (and hence the tumour itself). Although numerous studies have reported CTCs to be effective prognostic and predictive biomarkers in cancer patients these have been conducted almost exclusively on a retrospective basis. Few studies have been attempted where CTCs are utilised prospectively in patient stratification [27,37]. In summary, the IF methods developed in the present study are, to the best of our knowledge, the first capable of detection of MCT1 and MCT4 in CTCs and offer the potential to act as sensitive and selective biomarkers assays in the clinical evaluation of drugs targeting these proteins. As the field of cancer metabolism as a therapeutic target in drug design is currently growing in popularity, a broad application is envisaged for the new assays.

## Conclusion

Novel immunofluorescence methods have been developed for the visualisation of MCT1 and MCT4 in CTCs enriched in the Veridex™ CellSearch system using antibodies that have been optimised for selectivity. This methodology will be used to determine the expression levels of MCT1 and MCT4 in CTCs for the Phase I Clinical Trial of AZD3965, a selective MCT1 inhibitor. By correlating MCT1 expression levels in CTCs before and after AZD3965 treatment, the responsiveness of the patient can be determined thus providing a prognostic biomarker for disease progression. MCT4 expression levels in CTCs will also be taken into consideration as a biomarker for resistance to AZD3965 treatment in cells that retain the ability to export lactate during glycolysis.

## Abbreviations

CK: Cytokeratin
EpCAM: Epithelial cell adhesion molecule
MCT1: Monocarboxylate transporter-1
MCT4: Monocarboxylate transporter-4
CTC: Circulating tumour cell
SCLC: Small cell lung cancer
HNV: Healthy normal volunteer
BCA: Bichuronic acid
